# Deficiency of *P2RY11* causes narcolepsy and attenuates the recruitment of neutrophils and macrophages in the inflammatory response in zebrafish

**DOI:** 10.1007/s10565-024-09882-5

**Published:** 2024-05-21

**Authors:** Lin Zhao, Li-feng Wang, Yi-chen Wang, Ao Liu, Qian-wen Xiao, Ming-Chuan Hu, Ming-zhu Sun, Hui-yu Hao, Qian Gao, Xin Zhao, Dong-yan Chen

**Affiliations:** 1 Department of Histology and Embryology, School of Medicine, Nankai University, Tianjin, 300071 People’s Republic of China; 2 Institute of Robotics and Automatic Information System (IRAIS), Tianjin Key Laboratory of Intelligent Robotic (tjKLIR), Nankai University, Tianjin, 300350 China

**Keywords:** P2RY11, Narcolepsy, Inflammation, Neutrophil, Macrophage, Zebrafish

## Abstract

Purinergic receptor P2Y11, a G protein-coupled receptor that is stimulated by extracellular ATP, has been demonstrated to be related to the chemotaxis of granulocytes, apoptosis of neutrophils, and secretion of cytokines in vitro. P2Y11 mutations were associated with narcolepsy. However, little is known about the roles of P2RY11 in the occurrence of narcolepsy and inflammatory response in vivo. In this study, we generated a zebrafish *P2Y11* mutant using CRISPR/Cas9 genome editing and demonstrated that the *P2Y11* mutant replicated the narcolepsy-like features including reduced HCRT expression and excessive daytime sleepiness, suggesting that *P2Y11* is essential for HCRT expression. Furthermore, we accessed the cytokine expression in the mutant and revealed that the *P2RY11* mutation disrupted the systemic inflammatory balance by reducing *il4*, *il10* and *tgfb,* and increasing *il6*, *tnfa*, and *il1b.* In addition, the *P2RY11*-deficient larvae with caudal fin injuries exhibited significantly slower migration and less recruitment of neutrophils and macrophages at damaged site, and lower expression of anti-inflammatory cytokines during tissue damage. All these findings highlight the vital roles of P2RY11 in maintaining HCRT production and secreting anti-inflammatory cytokines in the native environment, and suggested that *P2RY11-*deficient zebrafish can serve as a reliable and unique model to further explore narcolepsy and inflammatory-related diseases with impaired neutrophil and macrophage responses.

**Graphical abstract:**

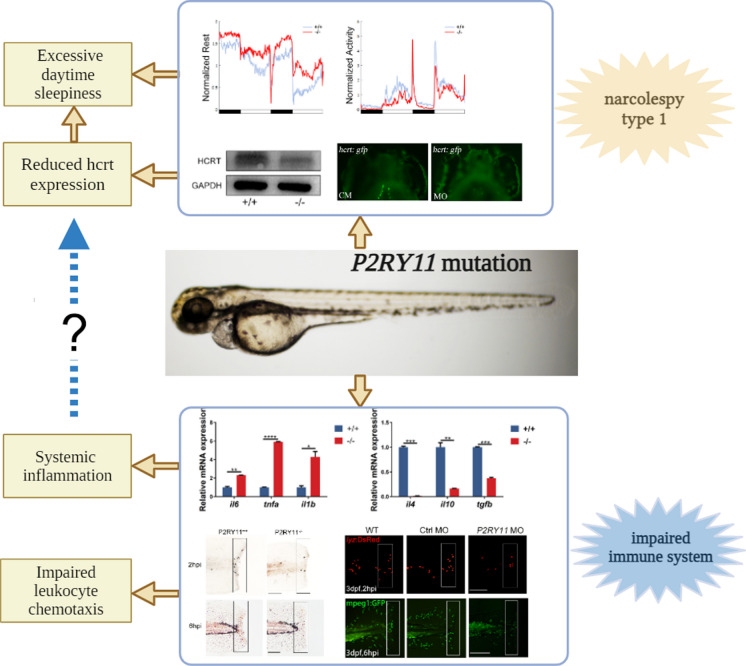

**Supplementary Information:**

The online version contains supplementary material available at 10.1007/s10565-024-09882-5.

## Introduction

Extracellular ATP released from cellular stress or apoptosis can be recognized as a damage-associated molecular pattern (DAMP) and can lead to the coordinated activation of P2 purinergic receptors on immune cells (Klaver et al. 2022). Purinergic receptor P2Y11, belonging to the P2 purinergic receptors family of G protein-coupled receptors (GPCRs), is expressed in diverse immune cell types, including macrophages, dendritic cells, and neutrophils (Klaver and Thurnher 2021). It could couple to extracellular ATP and translate the ATP alarm into a cytoprotective response via immunomodulation (Di Virgilio et al. 2020). Activation of the P2Y11 receptor could trigger intracellular cAMP signaling and induce downstream protein kinase C (PKC) (Klaver et al. 2022).

Previous studies have reported that P2Y11-mediated purinergic signaling is involved in inflammatory response. The stimulation of P2Y11 could increase the chemotaxis of granulocytes, inhibit the apoptosis of neutrophils, and extend their survival (Kennedy 2017). Suppression of P2Y11 could inhibit the differentiation of the THP-1 cell line into M1 macrophages and the secretion of IL-6 following LPS stimulation (Sakaki et al. 2013). Interestingly, in human M2 macrophages, P2Y11 plays a role in the anti-inflammatory response via inhibiting TLR4-driven TNF-α secretion (Gruenbacher et al. 2021). The activation of P2Y11 reduced the increase in TNF-α induced by radiation and increased IL-10 release in the blood (Swennen et al. 2008). Recent evidence shows that P2Y11 functions as a key receptor for macrophages, playing a vital role in regulating cytokine expression and inhibiting virus replication upon nucleotide interactions (Andersen et al. 2023). However, almost all studies on the function of P2Y11 have only been conducted in various cell lines because the *P2Y11* gene is absent in rodents. Thus, the function of P2Y11 in modulating the in vivo inflammatory response is still unknown.

SNP (rs2305795) of the *P2RY11* gene is found to be related to narcolepsy with cataplexy (Narcolepsy type 1, NT1), which is an immune-related disease characterized by a significant loss of HCRT (hypocretin neuropeptide precursor; also known as orexin) neurons (Kornum et al. 2011). Besides rs2305795, other missense mutations of *P2RY11* were also found in NT1 and led to a functional defect through both cAMP and Ca^2+^ signaling pathways (Degn et al. 2017). These studies have confirmed the correlation between the *P2RY11* gene and narcolepsy. However, due to the lack of suitable animal models that can mimic the *P2RY11* mutation in NT1, it has not been directly proven that the reduction of P2RY11 can lead to the occurrence of NT1.

Zebrafish (Danio rerio) has emerged as a unique model for studying sleep/wake disorders and inflammation due to its advantages of rapid ex utero development, transparent embryos, daytime activity, and an inflammatory response similar to mammals’ (Novoa and Figueras 2012). More importantly, *P2RY11* is present in the zebrafish genome and shows the same syntenic location as those of other mammalian species, implying that zebrafish *P2RY11* is indeed an ortholog of human *P2RY11* (Dreisig and Kornum 2016). Furthermore, the role of HCRT in promoting wakefulness was conserved between zebrafish and mammals.

To better evaluate the connection between the reduction of P2RY11 and NT1, as well as the effect of P2RY11 deficiency on the inflammatory response in vivo, we generated and characterized a zebrafish *P2RY11* loss-of-function mutant. Mutant larvae exhibited excessive daytime sleepiness and a lower level of HCRT expression, implying that zebrafish *P2RY11* mutants convincingly model human narcolepsy. Moreover, P2RY11 deficiency caused elevated levels of pro-inflammatory cytokines and reduced levels of anti-inflammatory cytokines. It also decreased the expression of genes that mediate the chemotaxis of neutrophils and macrophages, leading to diminished accumulation of macrophages and neutrophils at the injury site during non-infectious inflammation. These findings suggest important roles of P2RY11 in the inflammatory response in vivo.

## Materials and methods

### Zebrafish husbandry and morpholino injection

Adult zebrafish were raised at 28°C in a professional recirculating water system under a light-dark cycle (14-h light and 10-h dark) (Kimmel et al. 1995). Larvae were maintained in E3 medium with methylene blue. The following strains were used in this study: *Tg(hcrt:GFP)*, *Tg(lyz:DsRed)*, and *Tg(mpeg1:GFP)*. Zebrafish *P2RY11* (NM_001204454.1) MO (5′-TGCATAAACTGTCGTTCTTCATCTCT-3’) and mismatch MO (5′-TGGAAAAAGTGTCCTTGTTCATCTC-3’) were obtained from Gene-Tools, LLC. Approximately 0.6 ng of each was injected into one-cell stage embryos of *Tg(hcrt:GFP)*, *Tg(lyz:DsRed)*, or *Tg(mpeg1:GFP)* to generate the morphants.

### Generation of *P2RY11* mutant zebrafish

Mutagenesis was carried out using CRISPR/Cas9 technology, as previously described (Vejnar et al. 2016). Cas9 messenger RNA was synthesized using the mMESSAGE mMACHINE SP6 kit (Invitrogen) after the Cas9 plasmid was linearized. A mixture of sgRNA and Cas9 mRNA was injected into zebrafish embryos at the one-cell stage. The embryos were raised to adulthood as the F0 generation and crossed with wild-type (WT) zebrafish to obtain F1 generation embryos lacking *P2RY11*. Finally, an F1 founder line with an 8-base pair deletion in the *P2RY11* gene was obtained and used in subsequent experiments.

### Neutral red staining

A 2.5 ug/ml neutral red solution (Solarbio) was prepared using E3 solution containing 0.003% PTU. Zebrafish larvae at 3 dpf were immersed in the solution for 8 h at 28.5 °C in dark. Images were taken with a microscope. Quantification was performed manually.

### Sudan Black staining

Fixed larvae of *P2RY11*^*−8 bp*^ and siblings at 3 dpf were immersed in SB solution (G1691; Solabio) for 30 min followed by washing with 70% ethanol. Images were taken with a microscope. Quantification was performed manually.

### Tail fin amputation

The tail fin was amputated using *P2RY11*^*−8 bp*^ mutants and siblings, or MO-injected *Tg(lyz:DsRed)* and *Tg(mpeg1:GFP)*. The tail fin was amputated with a sterile scalpel on Petri dishes coated with 1.5% agarose under a stereomicroscope. Amputated larvae were maintained in the E3 medium. MO-injected *Tg(lyz:DsRed)* and *Tg(mpeg1:GFP)* larvae were anesthetized and observed under a fluorescence microscope for neutrophils at 2 h post-injury (hpi) and macrophages at 6 hpi. *P2RY11*^*−8 bp*^ and its siblings were used for Sudan black staining and neutral red staining. Regenerated tail fins of *P2RY11*^*−8 bp*^ and its siblings were amputated at 6 hpi, and then harvested for RNA extraction.

### RNA extraction and quantitative real-time PCR

*P2RY11*^*−8 bp*^ embryos and their siblings were harvested at 3 dpf. Amputated regenerated tail fins were harvested at 6 hpi. Total RNA was extracted using TRIzol reagent and then transcribed into cDNA with a HiFiScript cDNA Synthesis Kit (CWBIO, China). qRT-PCR was performed using a SYBR labeling system (CWBIO, China) on an Applied Biosystems system (Thermo Fisher Scientific, US). The relative expression was calculated using the 2-ΔΔCt method, normalized to the beta-actin level. The primer pairs were summarized in Table S1.

### RNA sequencing analysis

Total RNA was extracted from 50 embryos of *P2RY11*^*−8 bp*^ and siblings, quantified, and its integrity was checked (*n* = 3). The high-throughput RNA-sequencing (RNA-seq) analysis was performed on an Illumina HiSeq 4000 by Shanghai Aksomics Biotech in Shanghai, China. Briefly, RNA libraries were constructed and quantified with an Agilent 2100 Bioanalyzer and an ABI StepOnePlus RT-PCR system (Thermo Fisher, MA, USA). The raw trimmed reads were aligned to the zebrafish reference genome (GRCz11) using HISAT2 software (v2.1.0). The transcript abundance for each sample was estimated using StringTie (v1.3.3), and the FPKM values for gene and transcript levels were calculated using the R package Ballgown (v2.10.0). Differential expressed genes were identified based on the following criteria: fold change > 1.5 and *P*-value < 0.05.

### Sleep/wake behaviors

A video‐tracking system was modified from a previous study and was applied to record 48-h continuous sleep/wake behaviors (Rihel et al. 2010). 12 larvae of *P2RY11*^−8 bp^ and their siblings at 96 hpf were placed in a 96‐ well plate, with one larva in each well. 400 μL of E3 solution in each well can help maintain a nearly flat surface at the top of the wells, ensuring clear larval images with high resolution, brightness, and contrast. The 96‐well plate was illuminated with diffuse white light (7:30 AM to 9:30 PM) and constant infrared LED light. Images were recorded utilizing a video camera, which was equipped with a stationary megapixel lens (MP5018) and an infrared-penetrable filter, enabling the capture of infrared light. The entire system was kept at a steady temperature of 28°C and monitored for 48 h from 96 to 144 hpf.

### Western blot and antibodies

Zebrafish larvae at 5 dpf or adult tail fins were lysed in RIPA (CWBIO, China) with PMSF (Sigma-Aldrich). The proteins were quantified, and subjected to SDS-PAGE. They were then transferred onto a PVDF membrane. The primary antibodies are listed as follows: mouse anti- GAPDH (1:5000; Abmart, China), mouse anti- actin (1:1000; Millipore), rabbit anti- zHCRT (1:1000; produced by HuaBio, Hangzhou, China), and rabbit anti- zP2RY11(1:1000 produced by HuaBio, Hangzhou, China).

### Image acquisition and processing

The injured MO-injected *Tg(lyz:DsRed)* and *Tg(mpeg1:GFP)* larvae were mounted in 1% agarose after being anesthetized. Subsequently, the injured fin was imaged using a confocal microscope at 10 × . Images were taken every 3 min until 6 hpi for macrophages in *Tg(mpeg1: GFP)* or 2 hpi for neutrophils in *Tg(lyz:DsRed).* The images were used for analyzing the motility of macrophage and neutrophil with the software ImageJ (NIH, USA).

### Data analysis

All experiments were performed in triplicate. One-way ANOVA and unpaired two-tailed t-tests were used to evaluate differences among the various groups. Graph Prism 6 and MATLAB were utilized for statistical analysis and chart creation. The data is presented as the mean ± standard deviation (SD). Significance was set as *P* < 0.05 (*), *P* < 0.01 (**), and *P* < 0.001 (***). A phylogenetic tree was constructed using the neighbor-joining method with 1000 bootstrap replicates in MEGA 7.0. Heatmaps were generated with Heatmapper. Gene Ontology (GO) term enrichment analysis was conducted using the sources in the Metascape website.

## Results

### Establishment of *P2RY11* mutant

**Fig. 1:**
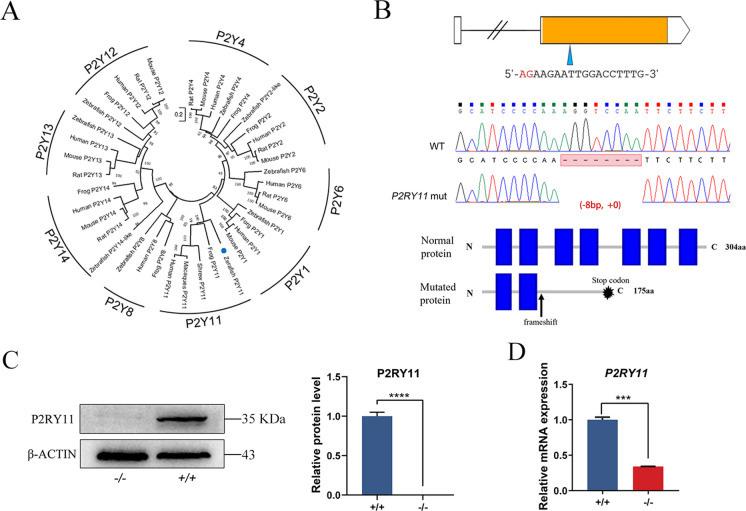
Phylogenetic trees of the P2YR family and the generation of a *P2RY11*^*−/−*^ mutation in zebrafish using CRISPR-Cas9 editing. A. Phylogenetic analysis was conducted on the predicted protein sequences of 8 families of P2Y receptors across 7 species using the neighbor-joining method. B. Structure of the zebrafish *P2RY11* gene and protein. A guide RNA was designed to specifically target exon 2. The *P2RY11*^*−/−*^ allele was created by deleting 8 bases in exon 2. The "-" indicates the deleted nucleotides. The mutation results in a frameshift and premature truncation of the protein. The blue boxes represent the transmembrane domains of P2RY11 protein. C. Western blot and statistical analysis (*n* = 3) of the P2RY11 protein in the tail of the *P2RY11*^−/−^ and *P2RY11*^+/+^ adult (3 mpf) zebrafish. D. The expression level of *P2RY11* mRNA in the 3-dpf *P2RY11*^−/−^ and *P2RY11*^+/+^ larvae was analyzed by qRT- PCR (*n* = 20). T-test, ***p < 0.001, **** p < 0.0001

To elucidate the relationship between zebrafish P2Y11 and its corresponding orthologues in different taxa, a phylogenetic analysis were performed using the protein sequences of 8 families of P2Y receptors across 7 species (shrew, zebrafish, human, macaques, rat, mouse, and frog) using the neighbor-joining method (Fig. 1A). The P2Y receptors can be categorized into two clades. The P2Y11 cluster forms one clade with clusters of P2Y1, P2Y2, P2Y4, and P2Y6. Clusters of P2Y8, P2Y12, P2Y13, and P2Y14 forms another clade. As expected, zebrafish P2Y11 is located in the cluster of P2Y11.

To investigate the role of *P2RY11* in sleep and inflammation, a *P2RY11*mutated zebrafish line was generated by targeting the exon 2 of *P2RY11* (Fig. 1B). A *P2RY11* mutation with an 8- bp deletion was identified, causing a frameshift mutation in the protein-coding region, which consequently triggered premature termination of translation. To verify the mutation, the expression of *P2RY11* mRNA and protein were measured in WT and *P2RY11* mutants. The results showed that the expression of *P2RY11* was decreased in mRNA level and undetected at the protein level in *P2RY11* mutants (Fig. 1C, D). Therefore, the generated *P2RY11* (-8 bp) mutant is a loss-of-function mutation.

### Morphological analysis of *P2RY11*-deficient zebrafish larvae

**Fig. 2:**
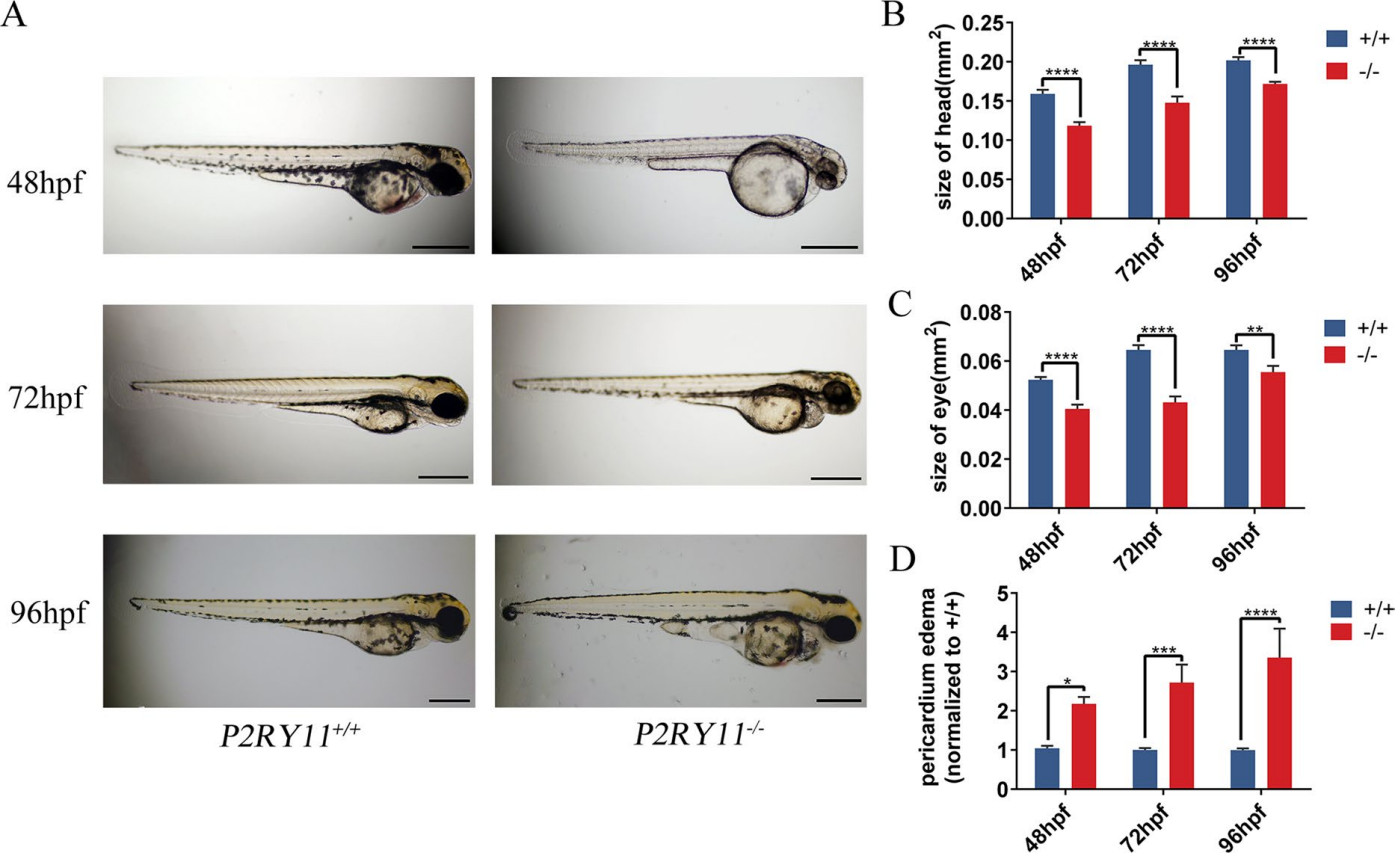
Morphological analysis of *P2RY11*^*−/−*^ zebrafish larvae. A, Morphology of *P2RY11*^−/−^ and *P2RY11*^+/+^ larvae at 48, 72, and 96 hpf. B-D, Statistical analysis for the head (B), eye (C), and pericardium (D) of *P2RY11*^−/−^ and *P2RY11*^+/+^ larvae at 48 hpf (*n* = 28), 72 hpf (*n* = 23), and 96 hpf (*n* = 28). *Scale bar*: 500 μm. T-test for analysis, *p < 0.05, ** p < 0.01 ***p < 0.001, ****p < 0.0001

To examine whether *P2RY11* deficiency would affect early development, we observed morphologic characteristics from 4-cell stage. No difference was found between the *P2RY11*^−8 bp^ mutant and its siblings from 4-cell stage to 24 hpf. With development progressing, the eyes and head of *P2RY11*^−8 bp^ larvae became significantly smaller than those of their siblings at 48, 72 and 96 hpf (Fig. 2A-C). Pericardial edema was also evident in *P2RY11*^−8 bp^ larvae (Fig. 2A). The size of the pericardium was significantly larger when compared with the control siblings at 48, 72, and 96 hpf (Fig. 2D). In summary, the deficiency of *P2RY11* could not affect the early embryonic development but impact later growth of the eye, head, and heart regions.

### Deficiency of *P2RY11* decreases the expression of HCRT

**Fig. 3:**
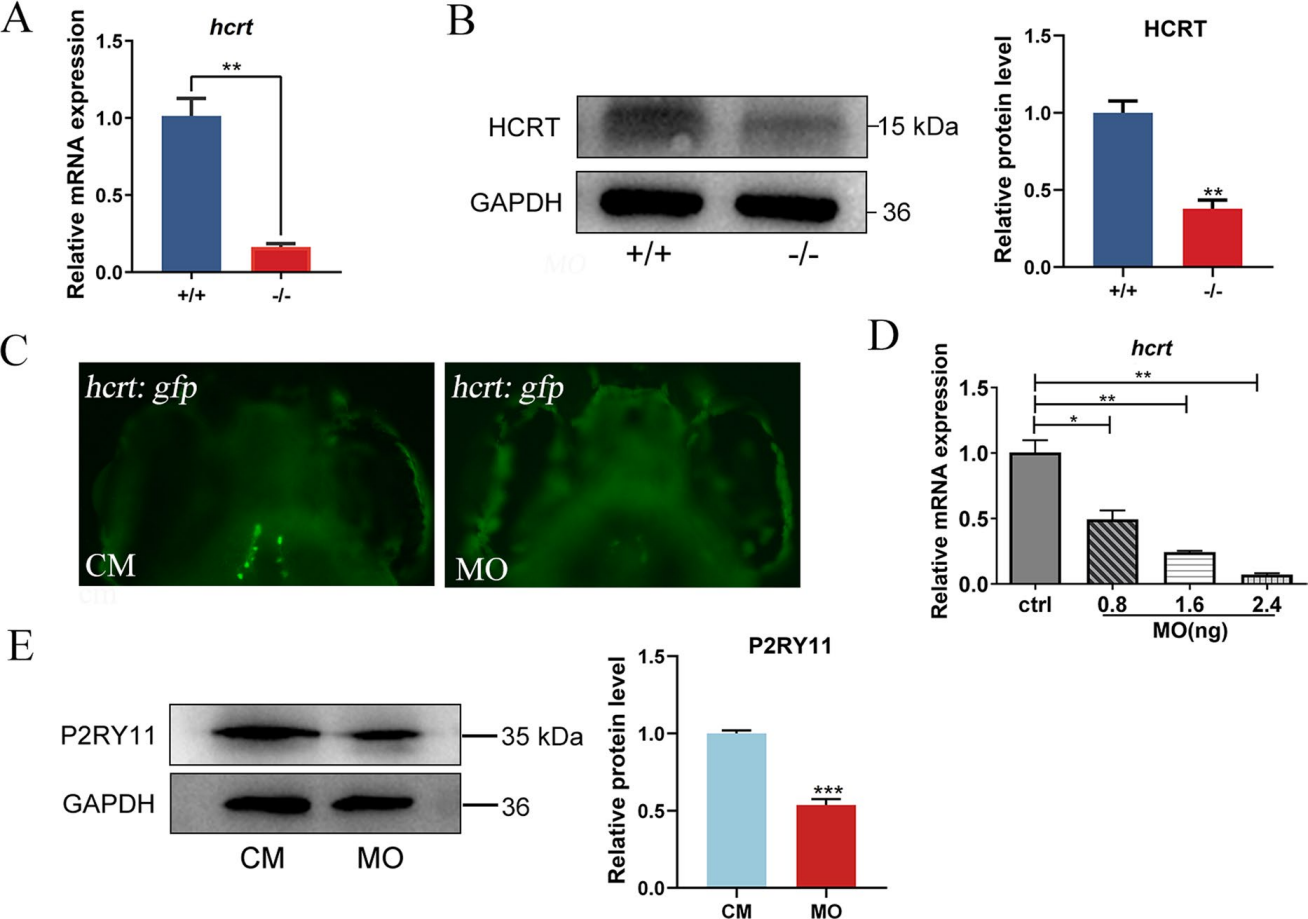
Deficiency of *P2RY11* reduces the HCRT expression. A, Reduced expression of *hcrt* mRNA in *P2RY11*^−/−^ mutants at 3 dpf detected by RT-qPCR (*n* = 20). B, Western blot (*n* = 10) and statistical analysis (*n* = 3) of the HCRT protein in *P2RY11*^−/−^ mutants at 3 dpf detected by western blot. C, Decreased HCRT neurons in *P2RY11* MO injected larvae at 3 dpf (*n* = 20). D, Reduced expression of *hcrt* mRNA in *P2RY11* MO morphants at 3dpf (*n* = 20). E, Western blot (*n* = 20) and statistical analysis (*n* = 3) of the P2RY11 protein in *P2RY11* MO morphants at 3dpf. T-test (A, B, E) and one way ANOVA (D) were conducted. *p < 0.05, **p < 0.01 ***p < 0.001

Given the association of *P2RY11* with NT1, the expression of HCRT was examined in *P2RY11-*deficient larvae at 3 dpf. Both *hcrt* mRNA and HCRT protein were reduced in* P2RY11*^−8 bp^ larvae compared to their WT controls (Fig. 3A, B). In order to investigate the impact of *P2RY11* deficiency on HCRT neurons, we knocked down the expression of *P2RY11* in *Tg(hcrt:GFP)* and observed a decrease in number of HCRT neurons and mRNA levels of *hcrt* in the *P2RY11* deficient larvae (Fig. 3C-E).

### Deficiency in *P2RY11* increases sleepiness and reduced waking activity during the daytime

**Fig. 4:**
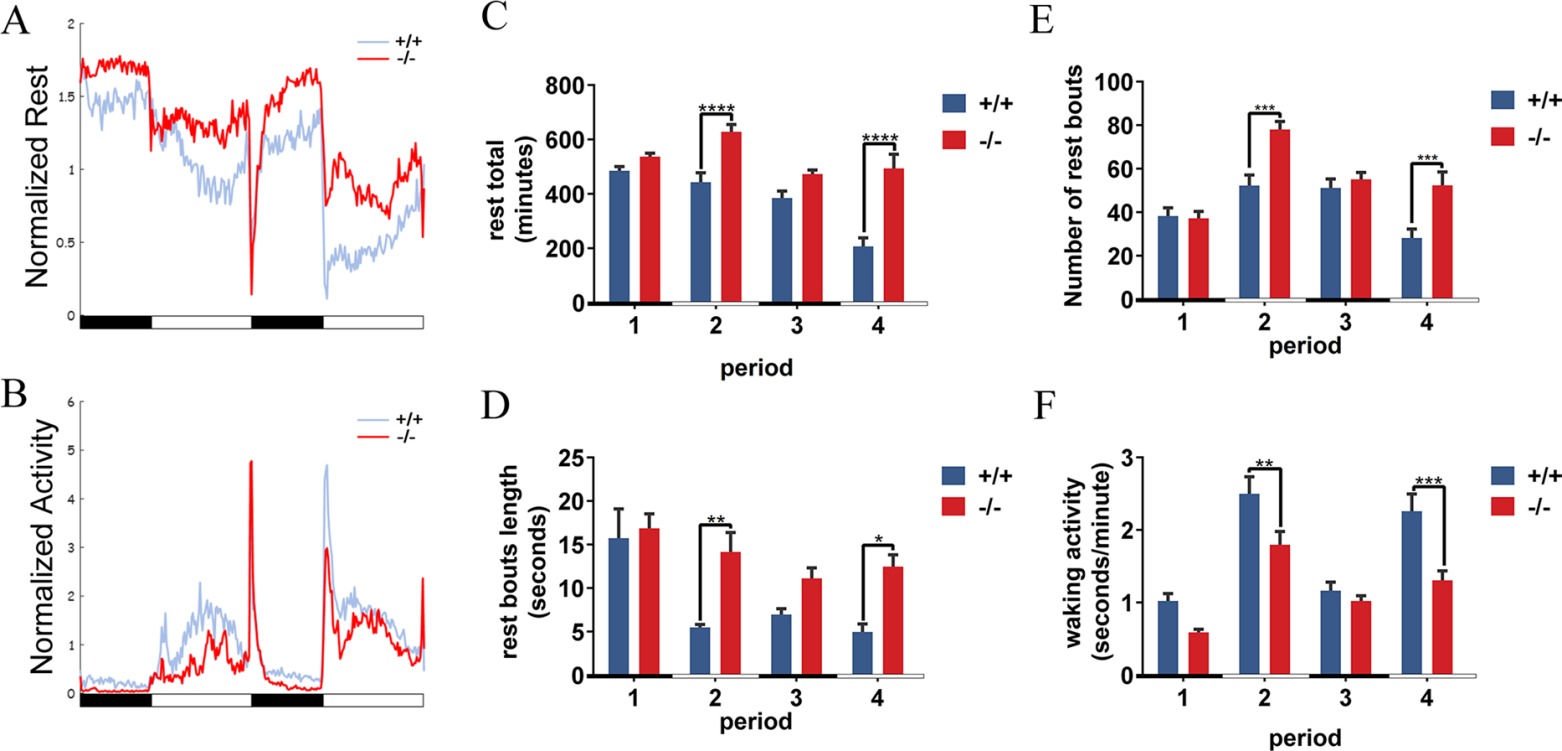
Abnormal sleep/wake pattern in *P2RY11*^−/−^ mutants. A, B, showing time sequence photos of rest total (A) and waking activity (B). The red line represents the mutant group, while the blue line indicates the WT group. C-F, Parameter analysis showed that rest time (C) and rest bout length (D) were prolonged in *P2RY11*^−/−^ mutants, with increased sleep frequency (E) during the daytime. Meanwhile, the waking activity (F) were decreased in the daytime recordings. The bars in black and white blow the x-axis indicate the tested nighttime and daytime periods, respectively. (t-test, *n* = 12, **p < 0.01, ***p < 0.001, ****p < 0.0001)

To examine whether the deficiency of *P2RY11* could affect the sleep patterns of zebrafish larvae, we conducted a sleep/wake analysis from 96 to 144 hpf. The results showed that *P2RY11*^−8 bp^ larvae exhibited reduced activity and increased rest during both night and daytime compared to their WT controls (Fig. 4A, B). The parameters regarding sleep/wake behavior were analyzed. *P2RY11* deficiency could significantly increase duration of rest, the number of rest bouts, and length of rest bouts only during the daytime period (Fig. 4C-E). Accordingly, both total activity and waking activity of *P2RY11*^−8 bp^ larvae were considerably lower than those of their WT controls in either daytime recorded (Fig. 4F; Fig. S1). Thus, *P2RY11*^−8 bp^ larvae exhibited a changed sleep/wake pattern similar to the excessive daytime sleepiness observed in narcolepsy patients.

### The lack of *P2RY11* affects the expression of genes involved in the immune system process

**Fig. 5:**
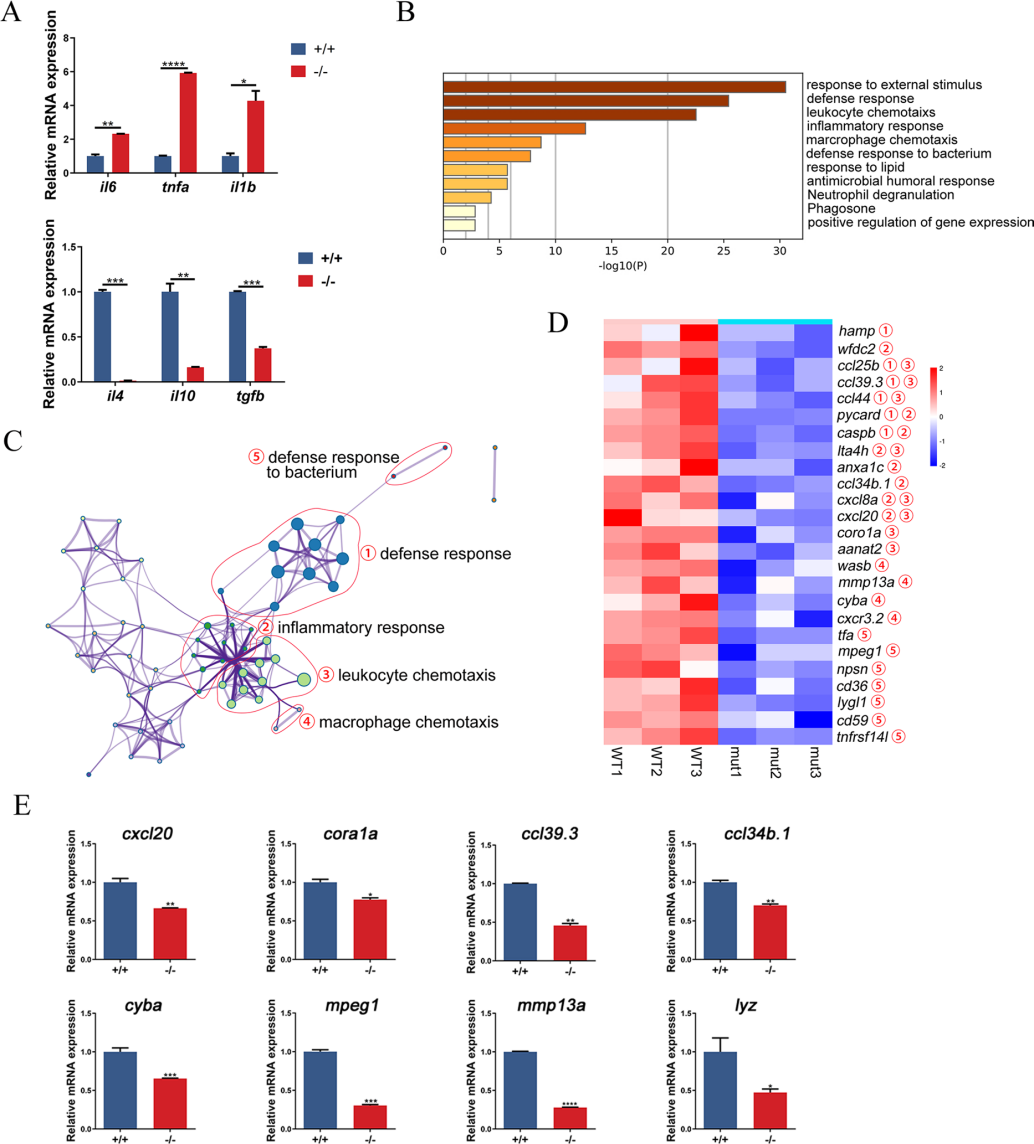
Effect of *P2RY11* mutation on the expression of genes related to the immune system process. A, Quantitative analysis of the expression of *il6*, *tnfa*, *il1b*, *il4*, *il10* and *tgfb*. (*n* = 20). B, Gene Ontology analysis of the DE genes related to the immune system process. C, The relationship of the enriched GO terms. D, Heatmap demonstrating downregulation of genes in *P2RY11*^−/−^ mutants involved in the main enriched terms (*n* = 3). E, qRT-PCR validation of the RNA-seq data. Downregulation of *cxcl20*, *cora1a*, *ccl39.3*, *ccl34b.1*, *cyba*, *mpeg1*, *mmp13a*, *lyz* (*n* = 20). T-test was performed, *p < 0.05, **p < 0.01, ***p < 0.001, ****p < 0.0001

Since *P2RY11* is expressed in different immune cell types, and its activation exerts both pro- and anti-inflammatory effect (Gruenbacher et al. 2021), here we conducted a qRT-PCR analysis to measure the mRNA levels cytokines to detect the impact of *P2RY11* deficiency on inflammatory status. Comparing with WT controls, the mRNA levels of *il6*, *tnfa*, and *il1b* were up-regulated, while the mRNA levels of *il4*, *il10* and *tgfb* were down-regulated in the *P2RY11*^−8 bp^ larvae (Fig. 5A).

To analyze the function of *P2RY11*, RNA sequencing analysis was performed and 1693 differential expressed (DE) genes were identified. GO term analysis were further conducted on DE genes using the Metascape database. The results showed that the plasma membrane signaling receptor complex (GO:0098802), calcium ion binding (GO:0005509), and protein refolding (GO:0042026) were the most enriched terms in the up-regulated DE genes (Fig. S2A, B). While metabolic processes (GO:008152), cellular locations (GO:0051179) and cellular processes (GO:009987) were the most enriched terms in the down-regulated DE genes (Fig. S2C). In addition, the immune system process (GO:0002376) was also enriched in down-regulated DE genes of *P2RY11*^−8 bp^ compared to WT controls (Fig. S2C). Based on the genes related to the immune system process, we further conducted GO term enrichment analysis and found that the defense response, leukocyte chemotaxis, and inflammatory response were the main enriched terms (Fig. 5B, C). Clustering analysis of the enriched GO term of the immune system process was conducted and showed that genes involved in neutrophil chemotaxis (c*xcl8a, cxcl20, ccl25b, ccl39.3, wasb, lta4h, ccl44*), inflammatory response (*pycard*, *caspb*, *lta4h*, *anxa1c*), defense response (*tfa, mpeg1, hamp, lygl1, npsn, tnfrsf14l, wfdc2*), and macrophage chemotaxis (*mmp13a, cyba, cxcr3.2*) were downregulated in* P2RY11*^−8 bp^ compared to their WT controls (Fig. 5D). The expression of *ccl34.b, coro1a, ccl39.3, cxcl20, cyba, mmp13a, lyz, and mpeg1* were detected to valid the RNA- seq data. It was found that mRNA levels of the selected genes were significantly reduced in *P2RY11*^−8 bp^ larvae, consistent with the RNA-seq results (Fig. 5E). Therefore, the above results show that the absence of *P2RY11* reduces the mRNA expression of several genes related to the chemotaxis of macrophages and neutrophils, inflammatory response, and defense response.

### The lack of *P2RY11* decreases the number of macrophages and neutrophils, as well as their accumulation at the injury site following fin amputation

**Fig. 6:**
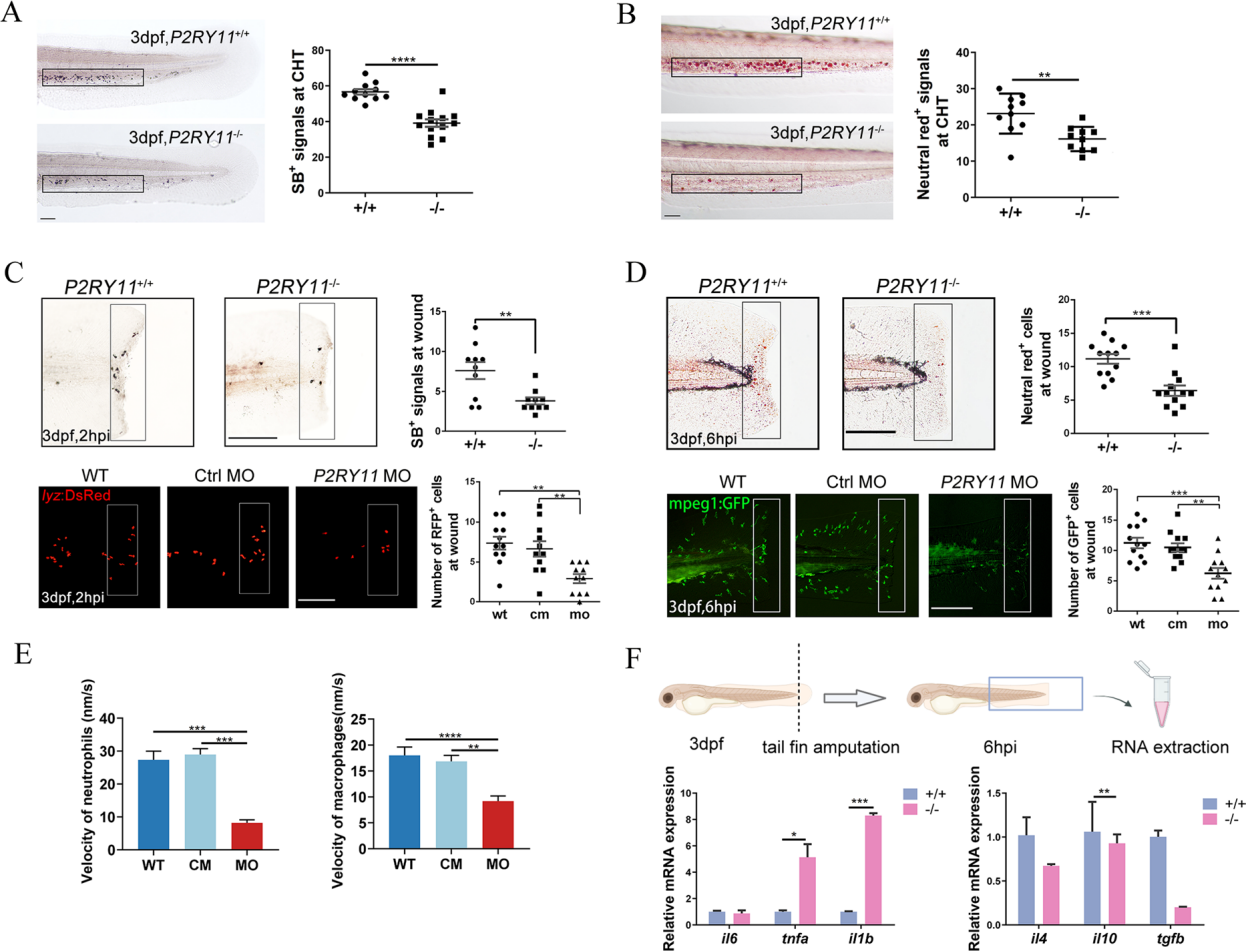
Deficiency of *P2RY11* reduced macrophages and neutrophils and their accumulation at the injury site following the fin amputation. A, The Sudan black B (SB) signal in siblings (upper) and *P2RY11* mutants (lower) at 3 dpf (t-test, *n* = 10). B, The neutral red signal in siblings (upper) and *P2RY11* mutants (lower) at 3 dpf in CHT (t-test, *n* = 10). C, recruited neutrophils to wounds following tail fin amputation. SB staining (upper) showed the recruitment of neutrophils at 2 hpi in 3dpf-siblings and *P2RY11* mutants (t-test, *n* = 10). SB^+^ cells were significantly reduced in *P2RY11*^−/−^ mutants in the quantified region (the black rectangle). The recruitment of neutrophils (RPF^+^ cells in the white rectangle) was also abolished in *P2RY11* MO morphants at 3 dpf in the transgenic *lyz:DsRed* compared with the control morpholino-injected group (cm) and wild type group (WT) (one-way ANOVA, *n* = 11). D, recruited macrophages to wounds following tail fin amputation. Neutral red staining revealed the recruitment of macrophages at 6 hpi in 3dpf-siblings and *P2RY11* mutants (t-test, *n* = 12). Neutral red^+^ cells were significantly reduced in *P2RY11* mutants within the quantified region (the black rectangle). The recruitment of macrophages (GFP^+^ cells in the white rectangle) was also abolished in *P2RY11* MO morphants at 3 dpf in the transgenic *mpeg1: GFP* compared with the control morpholino-injected group (CM) and wild- type group (WT) (one-way ANOVA, *n* = 12). E, Migration speed of neutrophils and macrophages in *P2RY11* knock-down larvae during the process toward the wound edge (one-way ANOVA, *n* = 5). F, Quantitative analysis of *il6*, *tnfa*, *il1b*, *il4*, *il10*, and *tgfb* from tails of WT and mutants at 6 hpi (t-test, *n* = 20). *p < 0.05, **p < 0.01, ***p < 0.001, ****p < 0.0001, *Scale bar*: 200 μm

Since *P2RY11*^−8 bp^ larvae showed decreased expression of *mpeg1* and *lyz*, which are specifically expressed in macrophages and neutrophils, respectively, we used Sudan Black staining and Neural red staining to label macrophages and neutrophils in *P2RY11*^−8 bp^ larvae. We found that mutation of *P2RY11* obviously reduced the numbers of neutrophils and macrophages in the caudal hematopoietic tissue (CHT) located at the ventral side of the tail (Fig. 6A, B).

Given the reduction of genes related to the chemotaxis of macrophage and neutrophil in *P2RY11*^*−8 bp*^ larvae, a caudal fin injury was induced to stimulate non-infectious inflammatory responses and access the chemotaxis of macrophages and neutrophils. The numbers of neutrophils and macrophages at the damaged site reached their peaks at 2 hpi and 6 hpi, respectively. Thus, larvae were collected at 2 hpi to detect the number of neutrophils in the cut region. SB staining revealed that depletion of *P2RY11* could significantly reduce the accumulation of neutrophils in the wound at 2 hpi, consistent with the findings in the *P2RY11*MO-injected transgenic line *Tg (lyz: Dsred)* (Fig. 6C). The macrophages were also examined in the cut region at 6 hpi in *P2RY11*^−8 bp^ larvae and *P2RY11*MO-injected transgenic line *Tg (mpeg1: GFP)*. Neutral red staining revealed that the number of macrophages at the wounded site of *P2RY11*^−8 bp^ larvae was conspicuously lower than the control group (Fig. 6D). Similarly, larvae injected with *P2RY11*MO displayed a reduced accumulation of macrophages in the cut region at 6 hpi compared with the WT groups (Fig. 6D). Additionally, the migration of macrophages and neutrophils was detected and found to be slower in *P2RY11* knocked-down larvae compared to WT larvae.

The cytokines in the tail at 6 hpi was also examined using qRT-PCR. It was found that *tnfa* and *il1b* was much higher in *P2RY11*^−8 bp^ larvae than in the WT group, whereas *il6* showed no difference between WT and *P2RY11*^−8 bp^ larvae. *Il4*, *il10*, and *tgfb* showed decreased expression in P2RY11^−8 bp^ larvae, especially conspicuously for *tgfb* expression. This suggests that the deficiency of *P2RY11* exaggerated inflammatory responses and affect the balance of the inflammatory response during tissue damage (Fig. 6E).

Taken together, the above results show that the depletion of *P2RY11* could affect the production of cytokines and the accumulation of the two types of leucocytes in the damaged tissue.

## Discussion

In this study, we generated a *P2RY11-*deficient zebrafish line and revealed that a *P2RY11* mutation exhibited similar features to NT1. Moreover, *P2RY11* deficiency significantly promoted a systemic inflammatory response and affected gene expression related to the recruitment of both neutrophils and macrophages. Furthermore, the *P2RY11* mutant exhibited deficiency in the accumulations of neutrophils and macrophages following tissue damage. All these findings highlight the significant roles of P2RY11 in maintaining HCRT expression and inflammatory response in vivo, which suggested that *P2RY11* mutated zebrafish can function as an animal model to further explore the narcolepsy and inflammatory-related diseases with impaired neutrophil and macrophage responses.

Although the association between *P2RY11* mutations or variants and NT1 has been confirmed in patients with narcolepsy, little is known about the function of P2RY11 in the development of the disease due to the absence of *P2RY11* gene in rodents. In this study, *P2RY11* mutant zebrafish exhibited the significant deformities such as microcephaly, microphthalmia, and pericardial edema initially at 48 hpf. Although the defects in *P2RY11*^*−/−*^ larvae became less prominent in adulthood, the morphological abnormalities of larvae still suggest that the P2RY11 gene may be involved in other aspects beyond sleep regulation. We also demonstrated the reduction of HCRT expression and excessive daytime sleepiness in zebrafish *P2RY11*^*−/−*^ mutants. Combined with the etiology and symptoms of NT1, it can be concluded that *P2RY11* deletion can cause narcolepsy-like symptoms by reducing the production of HCRT. Given the significance of HCRT production during NT1 development, the zebrafish *P2RY11*^−8 bp^ mutant serves as a dependable and distinctive model for NT1, which enables us to investigate HCRT abnormalities resulting from the mutant *P2RY11* for the first time.

Previous studies have suggested that P2Y11-dependent inflammatory signaling exhibits either proinflammatory or anti-inflammatory effects depending on the specific in vitro conditions. In our study, we observed an increase in pro-inflammatory cytokines and a decrease of anti-inflammatory cytokines in the native environment of *P2RY11*^*−/−*^ mutants. Both IL-4 and IL-10 are crucial anti-inflammatory cytokines that inhibit the release of TNF-α, IL-1β, and IL-6 in monocytes (te Velde et al. 1990, Zhang and An 2007). TGFb can counteract IL-1, IL-2, IL-6, and TNF, and shifting the state from active inflammation to resolution and repair (Zhang and An 2007). Therefore, the drastic decrease in anti-inflammatory cytokines in *P2RY11*^*−/−*^ mutants relieved their suppression on the secretion of pro-inflammatory cytokines, resulting in an increased level of pro-inflammatory cytokines. Together, it can be inferred that P2RY11 is necessary for anti-inflammatory cytokines, thus playing a crucial role in sustaining the balance of the immune system.

**Fig. 7:**
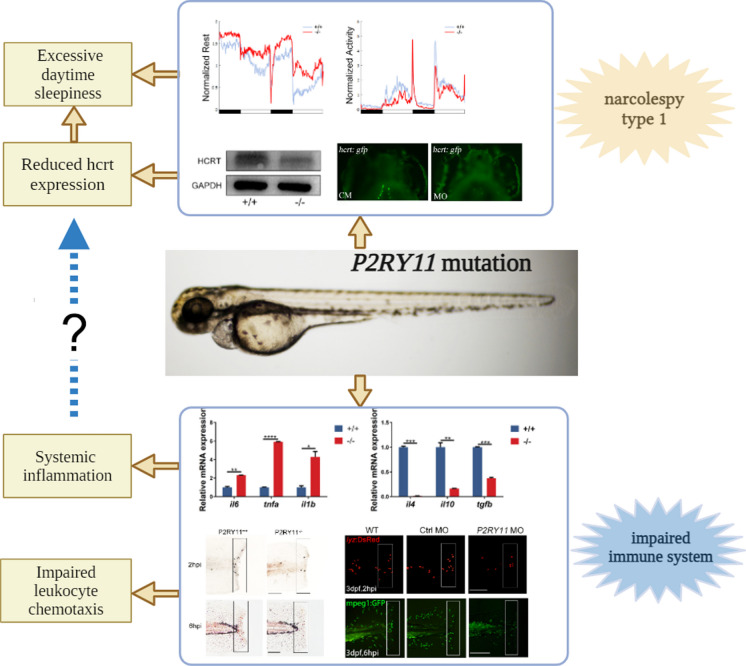
Diagram illustrating the effect of P2RY11 mutation on the immune response and the relationship of P2RY11 mutation with NT1

Systemic inflammation has been linked to decreased HCRT levels in patients (Ogawa et al. 2022). Increased serum TNF-a levels were found in narcolepsy patients (Chen et al. 2013). Combined with the extremely high expression of *il1b*, *il6* and *tnfa* found in zebrafish *P2RY11*^*−/−*^ mutants, we speculate that decreased HCRT expression may be due to the increased pro-inflammatory cytokines in the *P2RY11* mutant. This increase could trigger local inflammation in the brain (Fig. 7), ultimately leading to the cell death of HCRT neurons or the epigenetic silencing of *HCRT* (Seifinejad et al. 2023).

In addition to their important functions in innate immunity, macrophages and neutrophils contribute to wound healing and tissue regeneration (Li et al. 2012). Macrophages and neutrophils are recruited to sites of inflammation by gradients of chemoattractant (Kronlage et al. 2010). Here, we demonstrated that the slower migration and decreased accumulation of macrophages and neutrophils at the injury site, along with reduced expression of genes related with “leukocyte chemiotaxis” in the *P2RY11*^*−/−*^ mutant, indicate that the participation of P2RY11 is crucial for neutrophils and macrophages to infiltrate the damaged tissue. Purinergic signaling plays important roles in regulating chemotaxis of neutrophils and macrophages along chemical gradients (Wang and Chen 2018; Desai and Leitinger 2014). Both cells can translate the sensing of a chemoattractant into gradient navigation by releasing ATP and the autocrinally stimulating purinergic receptors linked to their membrane protrusions (Wang and Chen 2018). P2Y receptors on macrophages and neutrophils play a critical role in ATP-responsive amplification of chemotaxis (Desai and Leitinger 2014). The relationship between *P2RY11* and chemotactic navigation is completely unknown. It is worth further investigation as it might reveal the mechanism by which P2RY11 reduce the recruitment of macrophages and neutrophils. Additionally, we also observed that the numbers of neutrophils and macrophages in *P2RY11*^*−/−*^ mutant CHT were significantly lower than wild-type larvae, suggesting a potential role of *P2RY11* in myeloid hematopoiesis. On the other hand, it may also be a reason for the decreased recruitment of the two types of cells to the injury site.

In summary, we observed striking similarities in the expression of HCRT and daytime sleepiness between the NT1 patients and P2RY11-deficient zebrafish. Knockout of P2RY11 in zebrafish resulted in systemic inflammation, suggesting an essential role of P2RY11 in regulating the inflammatory response under normal physiological conditions. In addition, we revealed the vital function of P2RY11 in recruiting macrophages and neutrophils to damaged tissues. Our data expands understanding of the roles of P2RY11 in the occurrence of narcolepsy and inflammatory response. Therefore, our model provides an exciting novel tool to unravel the pathogenesis mechanisms of NT1, highlighting its typical traits, and to explore the function of P2RY11-mediated purinergic signaling in the infiltration of neutrophils and macrophages into damaged tissues.

## Supplementary Information

Below is the link to the electronic supplementary material.

Supplementary file1 (DOCX 2511 KB)

## Data Availability

The data and materials of the study can be obtained from the corresponding author upon request. The RNA-seq data was submitted to the NCBI GEO repository with the accession number GSE264674.
