# Supplementary material for: Deficiency of P2RY11 causes narcolepsy and attenuates the recruitment of neutrophils and macrophages in the inflammatory response in zebrafish
Source: Cell Biol Toxicol. 2024 May 21;40(1):36. doi: 10.1007/s10565-024-09882-5 (PMC11108927; doi:10.1007/s10565-024-09882-5)
Supplement: Supplementary file 1 — Supplementary file1 (DOCX 2511 KB) [file 10565_2024_9882_MOESM1_ESM.docx]

***Supplementary Materials***

**Deficiency of P2RY11 causes narcolepsy and attenuates the recruitment of neutrophils and macrophages in the inflammatory response in zebrafish**

Zhao Lin^1†^, Wang Li-feng^1†^, Wang Yi-chen^1†^, Liu Ao^1^, Xiao Qian-wen^1^, Hu Ming-Chuan^1^, Hao Hui-yu^1^, Gao Qian^1^, Sun Ming-zhu^2^, Zhao Xin^2,*^, Chen Dong-yan^1,*^

^1^Department of Histology and Embryology, School of Medicine, Nankai University, Tianjin 300071, P.R. China.

^2^Institute of Robotics and Automatic Information System (IRAIS), Tianjin Key Laboratory of Intelligent Robotic (tjKLIR), Nankai University, Tianjin 300350, China.

^†^These authors contributed equally to this work.

*Corresponding author

Zhao Xin

E-mail: zhaoxin@nankai.edu.cn.

Chen Dong-yan

E-mail: [chendy@nankai.edu.cn](mailto:chendy@nankai.edu.cn). ORCID:0000-0002-4672-398X

**Table of contents list**

1. **Supplementary methods**
2. **Supplementary Table S1.**
3. **Supplementary Figure S1.**
4. **Supplementary Figure S2.**

**Supplementary methods**

**Sleep/wake behaviors**

A video‐ tracking system was modified from a previous study and was applied to record 48-hour continuous sleep/wake behaviors (Prober et al., 2006, Rihel et al., 2010). 12 larvae of *P2RY11^-8bp^* and their siblings at 96 hpf were placed in a 96‐ well plate, with one larva in each well. 400 μL of E3 solution in each well can help maintain a nearly flat surface at the top of the wells, ensuring clear larval images with high resolution, brightness, and contrast. The 96‐well plate was placed in the device and illuminated with diffuse white light from 7:30 AM to 9:30 PM and constant infrared LED light. The images were recorded utilizing a video camera (MV-VS078FM, Micro Vision, Japan), which was equipped with a stationary megapixel lens (MP5018) and an infrared-penetrable filter, enabling the capture of infrared light. The entire system was kept at a steady temperature of 28°C and monitored for 48 hours from 96 to 144 hpf.

The processing program was developed using Microsoft Visual Studio 2010 (Microsoft Corporation, Redmond, WA, USA) and OpenCV 2.4.3 (Intel Corporation, Santa Clara, CA, USA). It was based on an algorithm that subtracted the adjacent frames from the former ones. Any one minute with a total movement duration of less than 0.1 second was categorized as one minute of rest (Prober et al., 2006, Rihel et al., 2010). A rest bout refers to a consecutive minute of rest (Prober et al., 2006, Rihel et al., 2010). The number of rest bouts represents the number detected within a single day or night (Prober et al., 2006, Rihel et al., 2010). Length of the rest bouts means the average duration of rest minutes (Prober et al., 2006, Rihel et al., 2010). Total activity was defined as the average amount of detected activity in seconds, including all rest bouts (Prober et al., 2006, Rihel et al., 2010). Waking activity was defined as the total amount of detected activity, excluding all one minute periods of rest (Prober et al., 2006, Rihel et al., 2010).

References:

Prober, D. A., J. Rihel, A. A. Onah, R.-J. Sung and A. F. Schier (2006). Hypocretin/Orexin Overexpression Induces an Insomnia-Like Phenotype in Zebrafish. The Journal of Neuroscience 26(51): 13400-13410.

Jason Rihel, David A Prober, Alexander F Schier (2010a). Monitoring sleep and arousal in zebrafish. Methods Cell Biol. 2010:100:281-94. doi: 10.1016/B978-0-12-384892-5.00011-6.

Jason Rihel et. al. (2010b). Zebrafish Behavioral Profiling Links Drugs to Biological Targets and Rest/Wake Regulation Science 327, 348 (2010); DOI: 10.1126/science.1183090

Table S1. Primers for genotyping, gRNA synthesis and RT-qPCR in this study.

| purpose | Gene | Sequence (5’-3’) |
| --- | --- | --- |
| genotyping | *P2RY11* | TCCACGACTTCGGTTCCAG |
|  |  | TTTCAAATCGACCTTCTCCC |
| gRNA synthesis | *P2RY11*T67 | TAATACGACTCACTATAGGAGAAGAATTGGACCTTTGGTTTTAGAGCTAGAA |
|  | Universal P | AAAAGCACCGACTCGGTGCCACTTTTTCAAGTTGATAACGGACTAGCCTTATTTTAACTTGCTATTTCTAGCTCTAAAAC |
| RT-qPCR | *il-6* | Fw **–** GTCTGCTACACTGGCTACACTCTTC |
|  |  | Rv **–** CGTCCACATCCTGAACTTCGTCTC |
|  | *il-4* | Fw **–** TGCCAAGCAGGAATGGCTTTGAA |
|  |  | Rv **–** TGCAGTTTCCAGTCCCGGTATATG |
|  | *il1b* | Fw **–** TCTGATGAGATGGACTGT |
|  |  | Rv **–** GATGTGCTTCATTCTGTTC |
|  | *il10* | Fw **–** AGCACTCCACAACCCCAATC |
|  |  | Rv **–**AGCAAATCAAGCTCCCCCATA |
|  | *tgfb* | Fw **–** GTCCGAGATGAAGCGCAGTA |
|  |  | Rv **–** TCAAATGAGAGCCAGCGGTT |
|  | *tnfa* | Fw **–** GCTGGTGATGGTGTCTAGGAGGAA |
|  |  | Rv **–** CCCTGGGTCTTATGGAGCGTGAA |
|  | *P2RY11* | Fw **–** AAGCACGGAAAACAAGGGCTC |
|  |  | Rv **–** CAGCATCCCCAAAGG |
|  | *hcrt* | Fw **–** ATTCTCACTCTTGGCAAAC |
|  |  | Rv **–** TTCATAACTGTCCACATCCT |
|  | *β-actin* | Fw **–** TTCACCACCACAGCCGAAAGA |
|  |  | Rv **–** TACCGCAAGATTCCATACCCA |
|  | *gapdh* | Fw **–** TTGCCGTTCATCCATCTT |
|  |  | Rv **–** GTGCCATCAGGTCACATACA |
|  | *cxcl20* | Fw **–** TGCGCTGCTGTTTGGAATGA |
|  |  | Rv **–** CCCCATCTGCCCTTCAGTAG |
|  | *ccl34b.1* | Fw **–** GACAACTGTGGGTGAGGAGG |
|  |  | Rv **–** GGTGGTATCGTGGAAGTCACA |
|  | *cyba* | Fw **–** GCTTTCTTACATGCTGCGCT |
|  |  | Rv **–** GTCTGGGAGGTGGGTTTTGA |
|  | *ccl39.3* | Fw **–** CCACCAGTAGCTGTGCCAAAA |
|  |  | Rv **–** CGTTTAGACTTTTACTCTGCTGTCC |
|  | *mmp13a* | Fw **–** GCTTCTGGCCTGAGATTCCA |
|  |  | Rv **–** ACCTTTATCCATTTGCCTTATTCCT |
|  | *coro1a* | Fw **–** GACAGACGAGGCTCCATACG |
|  |  | Rv **–** GCAAACCAATCATCCGCCTC |
|  | *cart1* | Fw **–** TGTGGAGAAGAAGCTCGGC |
|  |  | Rv **–** GACAACTGCACAACTTCCCGA |
|  | *hmx3b* | Fw **–** CAGAACCGCAGGAACAAGTG |
|  |  | Rv **–** GTGGTAGATGACGGGCACAC |
|  | *cckar* | Fw **–** TGGCCTCACCTCACTTGAAC |
|  |  | Rv **–** AACAGCCCCACGATTACCAG |
|  | *celf6* | Fw **–** GGCATACACCCCTATCCAGC |
|  |  | Rv **–** TTGGCTGAGATGACGTTCCC |
|  | *gpx3* | Fw **–** AACCATAAACGGGACGCAGT |
|  |  | Rv **–** CAAGGAAAGCCGAGGATGGT |
|  | *dlk2* | Fw **–** GGAAAATGTGCCGAAAATGG |
|  |  | Rv **–** GTAAACATCTTTGTCGCAGAATCTT |


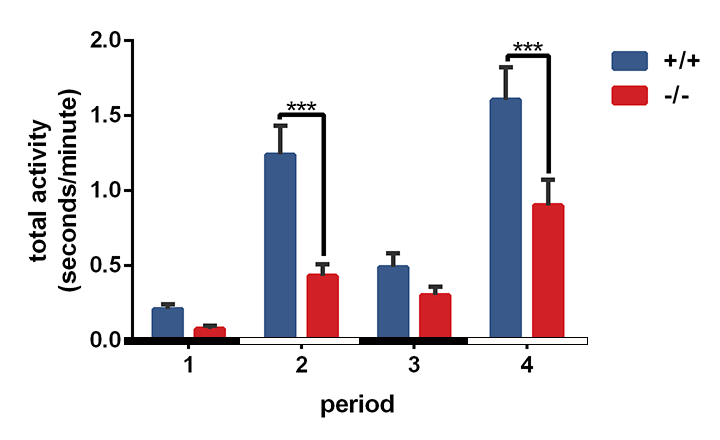


Figure S1. Parameter analysis for sleep/wake test showed the total activity was decreased in *P2RY11*^-/-^ mutants versus WT siblings, especially during daytime. n=12, ***p < 0.001.


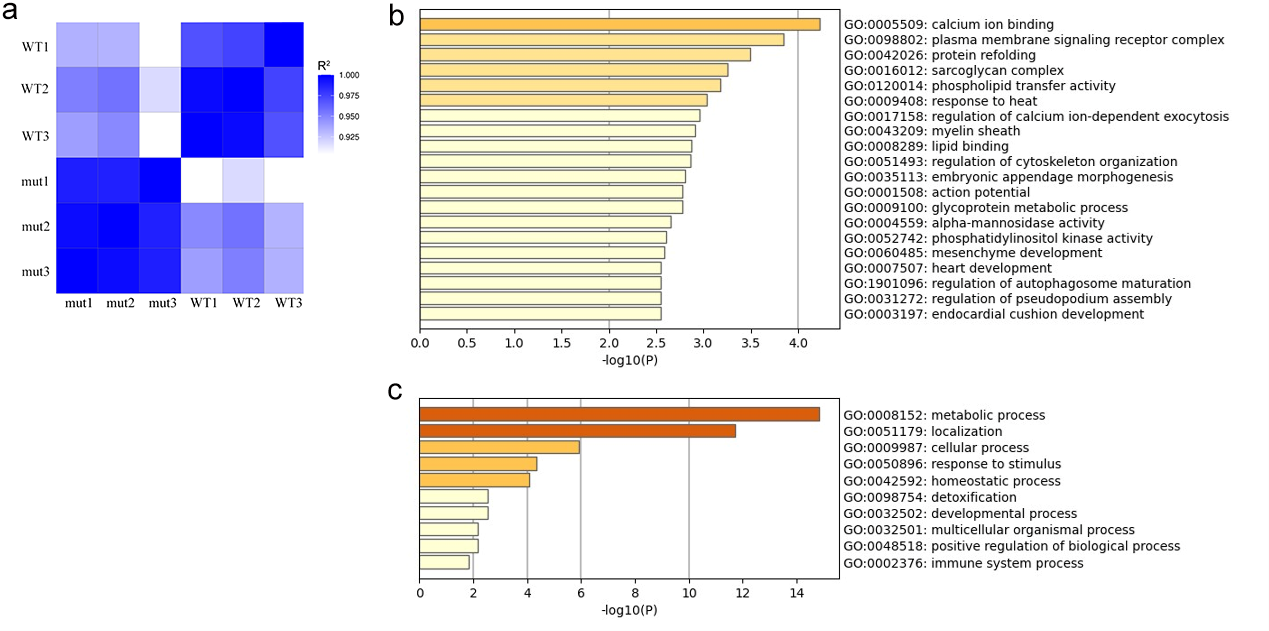


Figure S2. a, Distance heatmap analysis of WT siblings and *P2RY11*^-/-^ mutants. a, Color scale was shown on the right. The deeper the color, the smaller the difference between samples. b, GO analysis for the 1321 DE genes enriched in *P2RY11*^-/-^ mutants, with the color indicating the p-value. c, GO analysis for the 372 DE genes enriched in WT siblings, with the color indicating the p-value.
